# Correlation between peri-operative complication in middle ear cholesteatoma surgery using STAMCO, ChOLE, and SAMEO-ATO classifications

**DOI:** 10.1007/s00405-021-06679-8

**Published:** 2021-02-22

**Authors:** Tommaso Cacco, Stefano Africano, Gilda Gaglio, Luca Carmisciano, Enrico Piccirillo, Eolo Castello, Giorgio Peretti

**Affiliations:** 1 Department of Otorhinolaryngology-Head and Neck Surgery, IRCCS Ospedale Policlinico San Martino, University of Genoa, largo Rosanna Benzi 10, 16132 Genoa, Italy; 2 Department of Health Sciences, Section of Biostatistics, IRCCS Ospedale Policlinico San Martino, University of Genoa, 16132 Genoa, Italy; 3 Department of Otology and Skull Base Surgery, Gruppo Otologico, Piacenza, Rome, Italy

**Keywords:** Cholesteatoma, STAMCO, SAMEO-ATO, ChOLE, Labyrinthine fistula, Complications

## Abstract

**Purpose:**

To compare the relationship between the variable “complication” and the other variables of middle ear cholesteatoma classifications (STAMCO, ChOLE, and SAMEO-ATO).

**Methods:**

Retrospective study of 110 patients that underwent 132 middle ear surgeries between the 1 January 2012 and the 31 December 2019 for chronic otitis with cholesteatoma classified according to STAMCO, ChOLE, and SAMEO-ATO classifications in a tertiary health care centre.

**Results:**

Older age, male gender, STAMCO-T, and SAMEO-ATO [O_1_, T, O_2_, (s -)] and mastoid involvement (STAMCO-M and ChOLE-Ch) were associated with an increased risk of complication report.

**Conclusions:**

In our series, statistical analysis pointed out a relationship between surgical complications and age, gender, site, mastoidectomy type, and ossicular chain status at surgery. The choice of variables to be recorded for cholesteatoma staging should be carefully balanced, considering that “complication” variable could be a repetitive item.

## Introduction

Middle ear cholesteatoma is a non-neoplastic mass made of keratinizing squamous epithelium and keratin debris which accumulates in middle ear and mastoid cavities. The presence of cholesteatoma is associated with recurrent middle ear infections. Recurrent infections could lead to extracranial or intracranial complications. The formers are due to bony erosion and infection of noble structure, such as labyrinth or facial nerve, or accumulation of infectious material in mastoid or neck spaces. Infections spreading into intracranial spaces can cause epidural abscess, subdural abscess, purulent meningitis, brain herniation, brain abscess, or sinus thrombosis.

Many classifications for cholesteatomas and tympanomastoid surgery have been proposed with the aim of describe the severity of the cholesteatoma and the risk of recurrence and to compare surgical outcomes and surgical procedures [1–4]. The most recently proposed middle ear cholesteatoma classifications are STAMCO, ChOLE, and SAMEO-ATO classifications [2–6]. The presence of pre- or intra-operative complications is a variable considered both in STAMCO and ChOLE classifications.

The aim of this study is to describe the experience of our centre focusing on intra-operative complications according to STAMCO, ChOLE, and SAMEO-ATO classifications. Our goal is to analyse the relationships between the variable “complications” and the other variables of each classification.

## Materials and methods

### Patients

All procedures considered in this study were in accordance with the ethical standards of 1964 Helsinki Declarations and its later amendments. We retrospectively analysed surgeries performed in adult patients (>18 years age) for middle ear pathology at our ENT Surgery Department from 1 January 2012 to 31 December 2019. Patients without a histological diagnosis of middle ear cholesteatoma were excluded from analysis. Cases affected by congenital cholesteatoma were excluded. Clinical information and imaging studies were reviewed. Our cohort was composed by 110 patients, 54 males and 56 women, which underwent altogether 132 surgeries. 92 patients were operated once, 12 patients were operated two times, and 4 patients were operated three times. The reasons for subsequent surgery were cholesteatoma recurrence (94%) and tympanic membrane perforation (6%). Mean age was 54.09 ± 19.95 years. Median follow-up was 17 months (inter-quartile range, IQR, 3–34 months). 135 surgeries for middle ear cholesteatoma were collected, and surgical reports were revised and classified according to STAMCO, ChOLE, and SAMEO-ATO classifications. We considered as a complication pre- or intra-operative complications related to disease growth.

### Middle ear cholesteatoma classifications

STAMCO classification of middle ear cholesteatoma is a novel tool that describes the severity of the pathology focusing on the risk of recurrence depending on intra-operative findings, such as middle ear subsites involved by cholesteatoma, presence of complications, and ossicle state. These variables were chosen to represent the extension of the pathology, surgical complications, and difficulty to achieve complete removal. STAMCO-S, T, A, and M refer to different middle ear subsites, whereas STAMCO-C refers to the presence of complication and STAMCO-O to ossicular chain ossicle missing. Middle ear subsites which were considered are: difficult access sites, namely supratubal recess and sinus tympani (STAMCO-S), tympanic cavity (STAMCO-T), attic (STAMCO-A), and mastoid and antrum (STAMCO-M).

ChOLE classification of cholesteatoma considers middle ear subsites involved by cholesteatoma, ossicle status at the end of surgery, surgical complication, and mastoid pneumatization as indirect sign of Eustachian tube function. ChOLE-Ch refers to middle ear cholesteatoma extension; ChOLE-O classification represents the Austin–Kartush and Fisch classifications of ossiculoplasty; ChOLE-L reports the presence of life-threatening complications; ChOLE-E stands for Eustachian tube function, estimated with mastoid pneumatization degree at preoperative CT scan.

SAMEO-ATO classification summarizes relevant surgical details for middle ear surgery. SAMEO-ATO-S represents the stage of surgery (primary or revision surgery); SAMEO-ATO-A_1_ refers to the type of approach (retroauricular, transcanalar, and endaural); SAMEO-ATO-M records the type of mastoidectomy performed; SAMEO-ATO-E represents the surgical necessity of perform a reconstruction of the external ear after cholesteatoma removal; SAMEO-ATO-O_1_ refers to obliteration of mastoid cavity; SAMEO-ATO-A_2_ refers to the necessity of external ear canal widening; SAMEO-ATO-T records the type of tympanic membrane graft performed; SAMEO-ATO-O_2_ refers to the type of ossicular chain reconstruction performed.

### Statistical methods

Categorical variables were reported with count and percentages; continuous variables with mean and standard deviation (SD), median, and inter-quartile range (IQR). Chi-squared test was used to evaluate the associations between categorical variables. A logistic regression model was used for multivariable analysis, using the presence of peri-operative complications as dependent variable. For modelling purpose, the classification items were binned into binary variables accordingly to the number of subject and the severity of each variable level.

Bidirectional stepwise regression and Lasso regression with tenfold cross-validation procedure for the lambda parameter optimization were used to identify the most useful complication predictors.

No information rate (NIR) was used to indicate the accuracy of the model aware only of the most frequent outcome. Accuracy was used to report the proportion of complications correctly classified by the final model. The negative predictive value (NPV) indicates the proportion of cases predicted as not likely to develop complications who actually has not developed complications, and the Positive predictive value (PPV) indicates the proportion of cases predicted as likely to develop complications who actually had complications. *p *values below 0.05 were considered significative.

R-software version 3.6.0 was used for all statistical analyses, and Glmnet package [7] version 2.0-18 was used for the Lasso implementation.

## Results

**Fig. 1:**
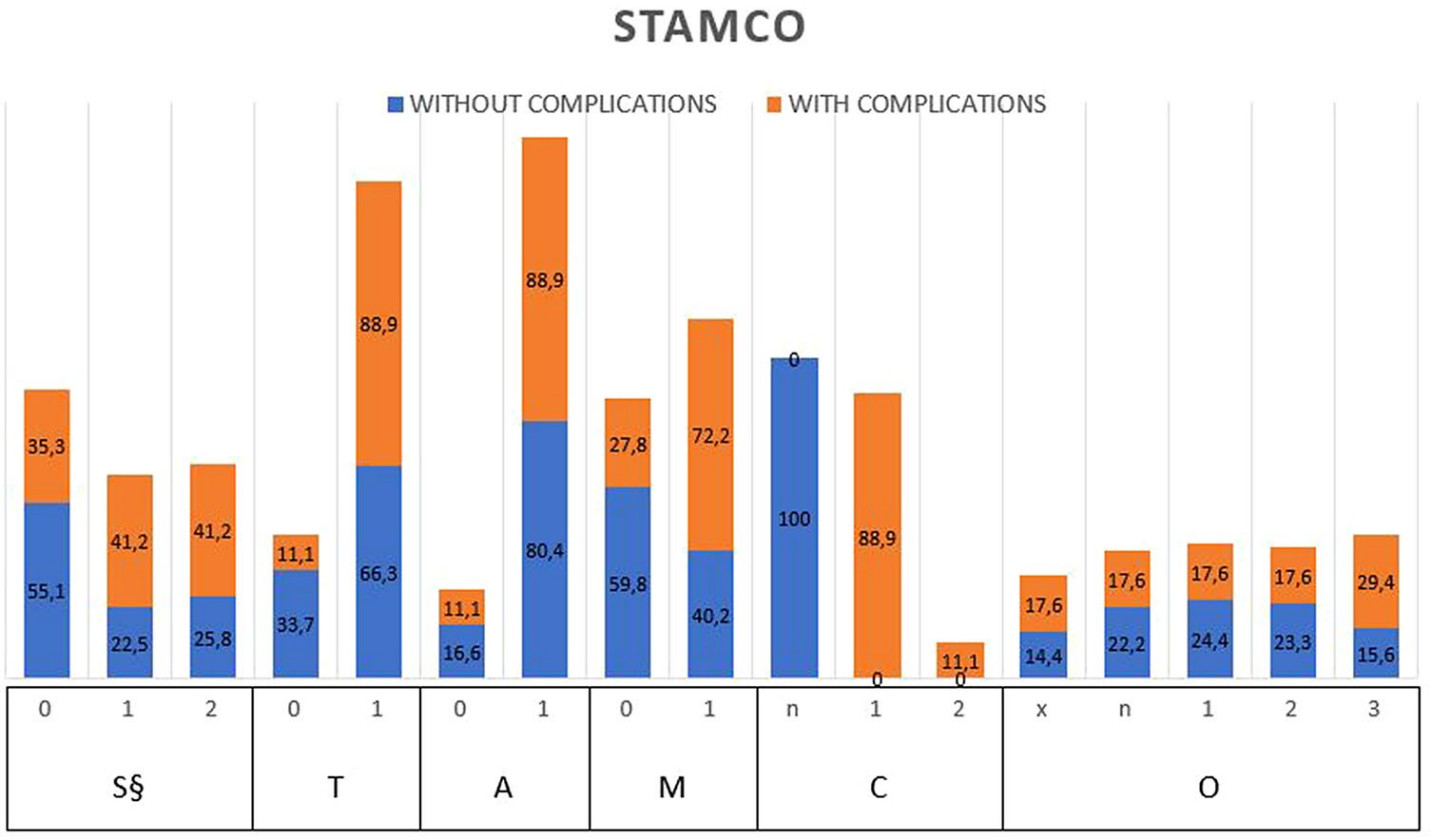
Cholesteatoma characteristics classified sec. STAMCO classification associated with peri-operative complications in patient’s cohort (percentage). STAMCO CLASSIFICATION S. §: Involvement of difficult access sites (supratubal recess, sinus tympani), T: involvement of tympanic cavity, A: involvement of attic/epitympanic space, M: involvement of mastoid and antrum, C: complication status, O: ossicular status at the beginning of the surgery, § 3 patients had both *S* = 1 and *S* = 2

**Fig. 2:**
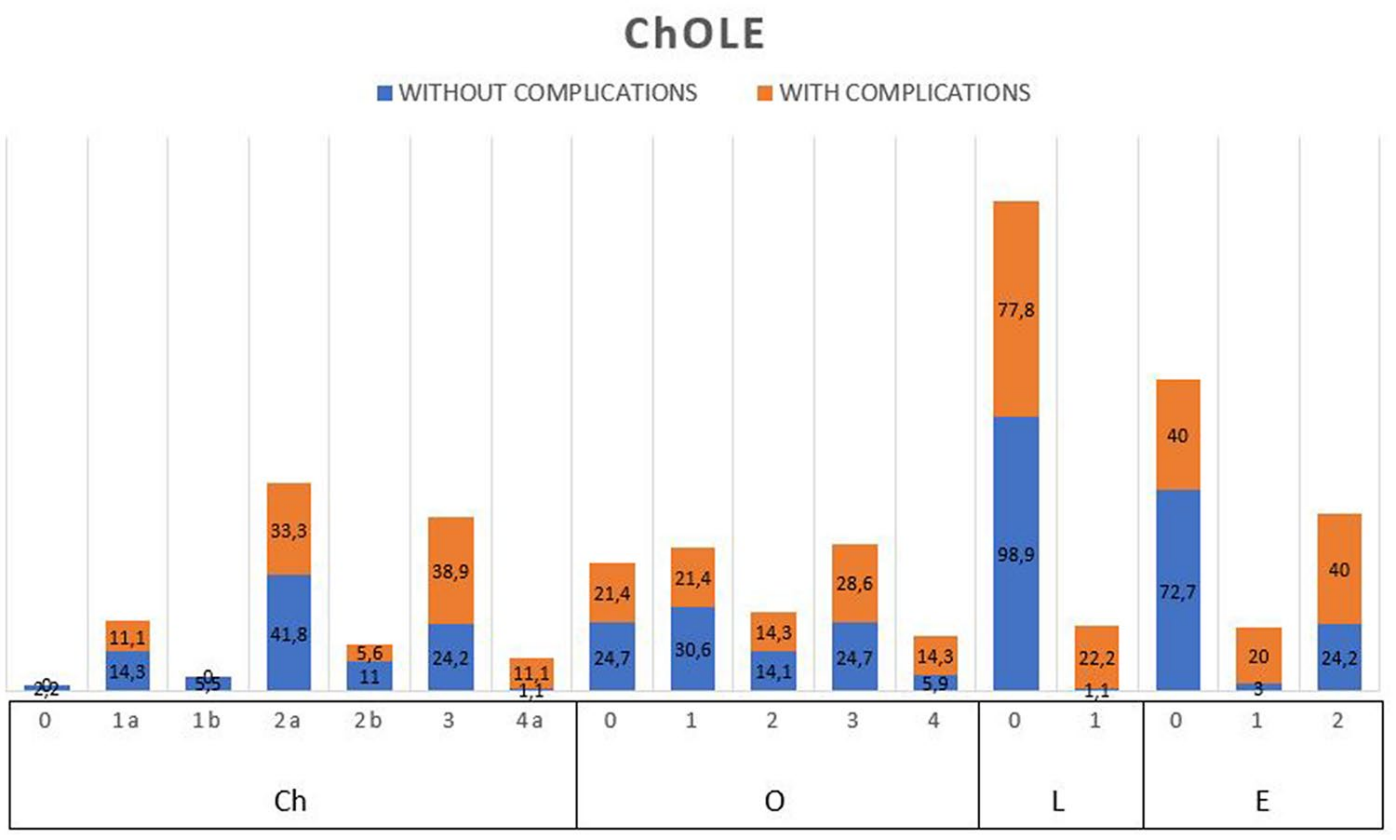
Cholesteatoma characteristics classified sec. ChOLE classification associated with peri-operative complications in patient’s cohort (percentage). ChOLE CLASSIFICATION. Ch: cholesteatoma extension, O: ossicular chain status at the end of the surgery, L: life-threatening complications, E: Eustachian tube disfunction

**Fig. 3:**
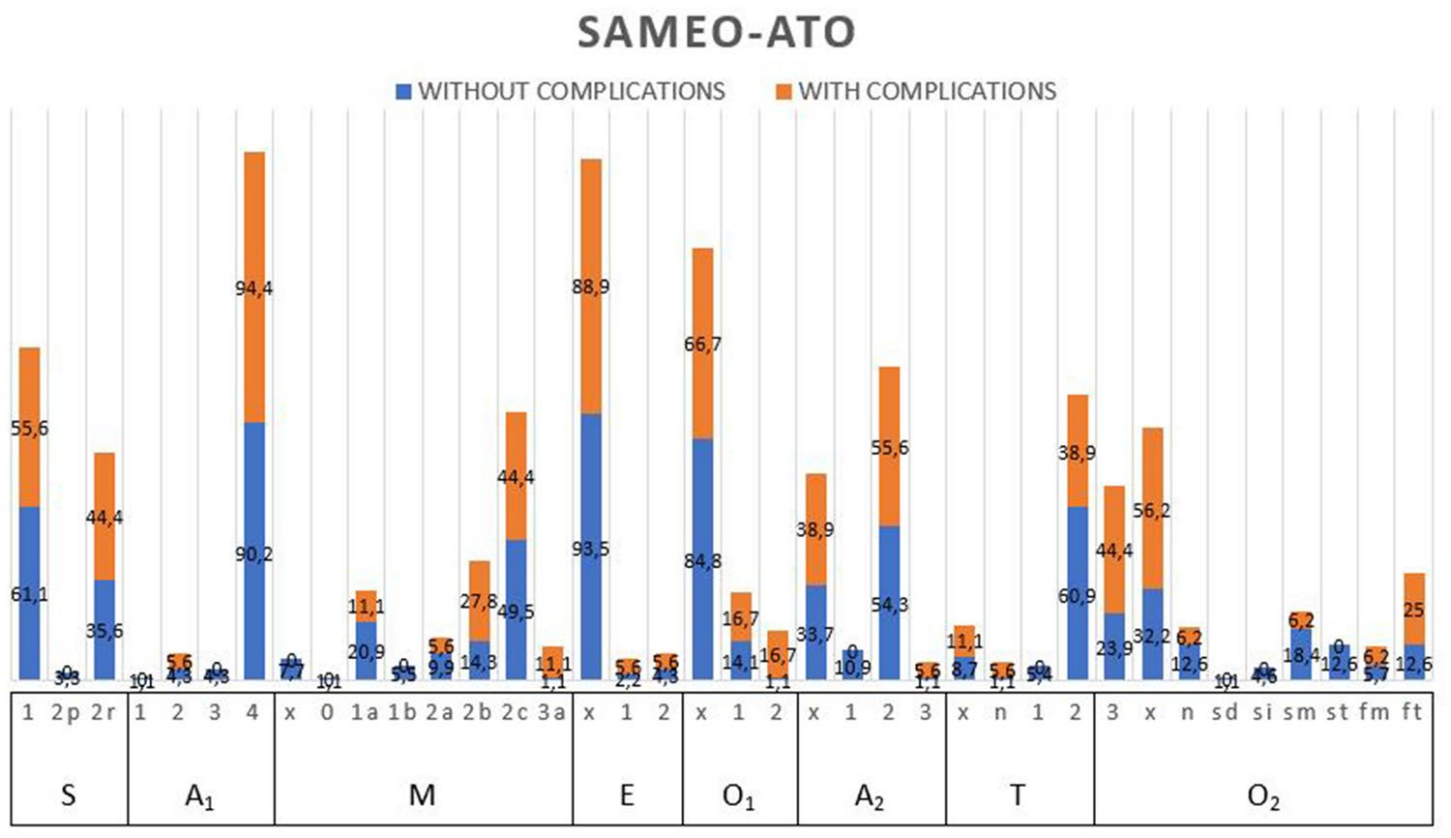
Cholesteatoma surgery characteristics classified sec. SAMEO-ATO classification associated with peri-operative complications in patient’s cohort (percentage). SAMEO-ATO CLASSIFICATION. S: stage of surgery, A_1_: type of approach, M$: mastoidectomy, E: external ear canal reconstruction, O_1_: obliteration of mastoid cavity, A_2_: access to middle ear (canalplasty), T: tympanic membrane reconstruction, O_2_: ossicular chain reconstruction performed, $ 6 patients had 1a + 2a and 3 had 1b + 2a

General patients’ characteristics are reported in Table 1. In our analysis, male patients had presented a non-significative trend of higher complications compared to females (*p* = 0.059). Elderly patients were significatively associated with complications (mean difference = -16.6 (95% CI -24.6, -8.8), *p* = 0.004). The optimal age cut-off of 49 years was detected using the Youden index; area under the ROC curve (AUC) was 0.746 (95% CI 0.628, 0.864). Overall, 18 patients underwent surgery more than once, but neither the number of recurrence nor the time from previous surgery appeared to be associated with a higher peri-operative complications rate. Cholesteatoma characteristics classified according to STAMCO and ChOLE classifications are plotted in Figs. 1 and 2. Surgeries characteristics according to SAMEO-ATO classification are plotted in Fig. 3. Surgical complication rates are reported in Table 2. In patients having more than one surgery, number of recurrence and time from previous surgery resulted not correlated with an increased frequency of intra-operative complications.

**Table 1:** General patients’ characteristics

		Overall *N* = 110	Patients with no surgery complications *N* = 92	Patients with any surgery complication *N* = 18	*p* value
No. of surgeries, N (%)	1	92 (83.6)	76 (82.6)	16 (88.9)	0.560
	2	14 (12.7)	13 (14.1)	1 (5.6)	
	3	4 (3.6)	3 (3.3)	1 (5.6)	
Gender, N (%)	M	54 (49.1)	41 (76)	13 (24)	0.059
	F	56 (50.9)	51 (91)	5 (9)	
Age, years Mean (SD) Median [IQR]		53.17 (19.73) 56.72 [36.32, 70.11]	50.45 (19.59) 52.76 [32.28, 68.34]	67.11 (13.99) 69.40 [56.20, 78.82]	0.004
Time from first surgery, months Mean (SD) Median [IQR] {*N*}		70.59 (57.98) 55.93 [36.45, 90.08] {4}	87.89 (56.99) 69.50 [55.93, 110.65] {3}	18.70 (–) 18.70 [18.70, 18.70] {1}	–
Time from previous surgery, months Mean (SD) Median [IQR] {*N*}		34.04 (26.96) 29.22 13.58, 43.12] {18}	36.54 (27.64) 33.55 [13.92, 44.02] {16}	14.10 (1.79) 14.10 [13.47, 14.73] {2}	0.261

**Table 2:** Pre- and intra-operative complications

Complication type	No. of complicated surgery *N* = 22	Frequency among complicated surgery (%)	Overall frequency (%)
Lateral semi-circular canal fistula	10	41.7	7.6
Middle cranial fossa fistula	6	25	4.5
Superior semi-circular canal fistula	2	8.3	1.5
Cutaneous-mastoid fistula	2	8.3	1.5
Non specified semi-circular canal fistula	2	8.3	1.5
Zygomatic abscess	1	4.2	0.8
Otoliquorrhea	1	4.2	0.8

Overall complication rate was 17% (95% CI 11–24). This finding was comparable to that published in the literature up to now [6]. The most common complication was lateral semi-circular canal fistula (LSC fistula) with a frequency of 41% among complicated surgery and an overall frequency of 7.6%. Other reported complications were middle cranial fossa fistula (overall frequency 4.5%), labyrinthine fistula, superior semi-circular canal fistula (SSC fistula), cutaneous-mastoid fistula (overall frequency 1.5%), zygomatic abscess, and otoliquorrhea (overall frequency 0.8%).

High association was detected among many overlapping items of the different classification systems (like ChOLE-O and STAMCO-O or SAMEO-ATO-O_2_, ChOLE-Ch and STAMCO-S,M, or SAMEO-ATO-M) but also within the same classification system for STAMCO (M, A) and SAMEO-ATO-(T, O_2_, S) but not for ChOLE.

**Fig. 4:**
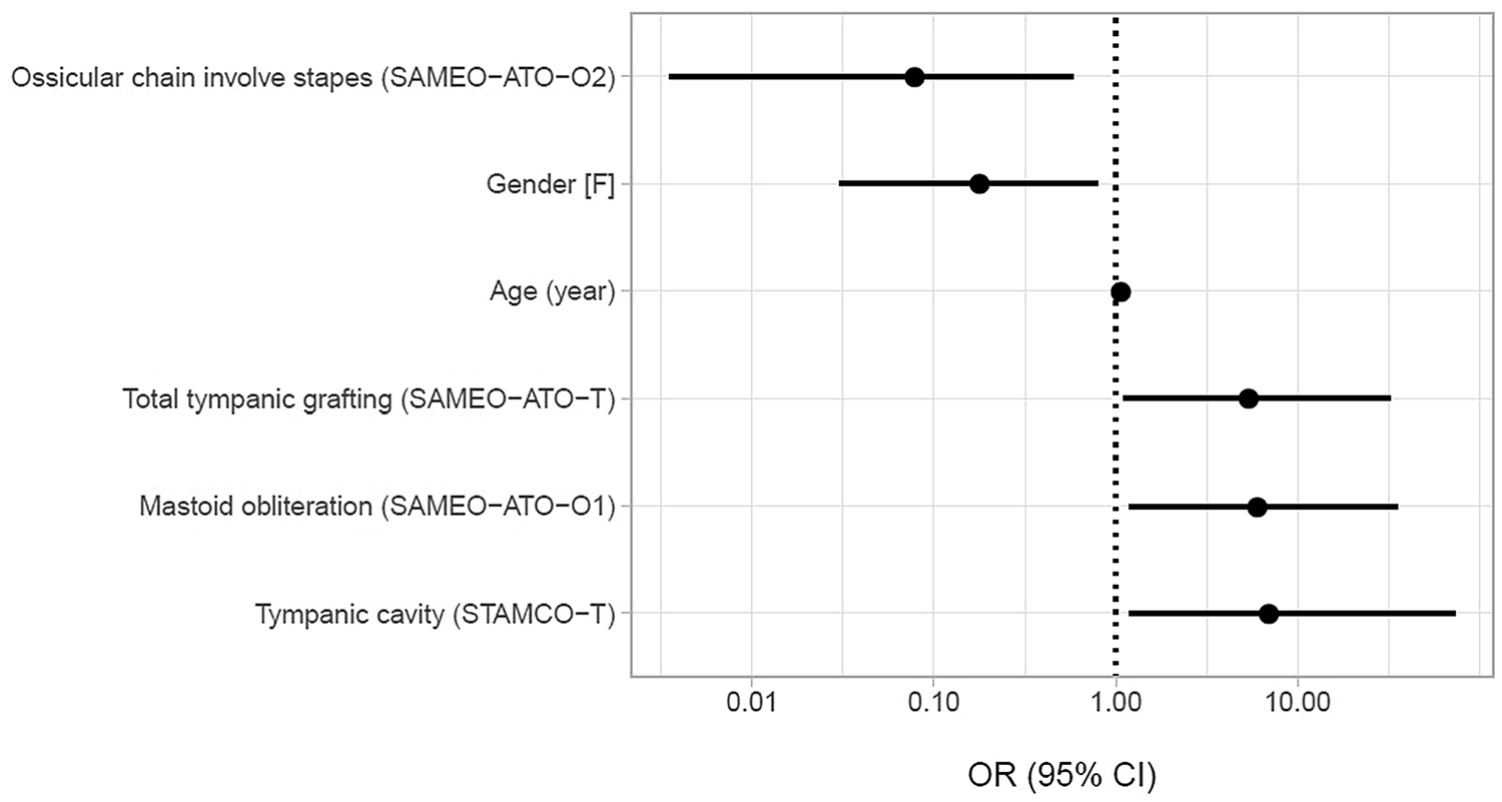
Forest plot of best model predictors

When used together, the most useful information to predict the development of complications was age, gender, STAMCO-T, and SAMEO-ATO [O_1_, T, O_2_, (s -)] (Table 3, Fig. 4). Older patients were more likely associated with complications compared to youngers (about 7% more frequently for each year of age). Females had significatively lesser complication rate than males (about 82% less frequently). Tympanic cavity involvement (assessed by STAMCO-T) was associated with higher complication rate than patients with cholesteatoma sparing tympanic cavity (about 7 times more frequently). Patient which underwent a total or subtotal tympanic membrane grafting (assessed by SAMEO-ATO-T) had significative higher complication rate than patients with other tympanic membrane perforations (about 5.4 more frequently). Mastoid obliteration (assessed by SAMEO-ATO-O_1_) was associated with higher complication rate compared to patients who had not undergone obliteration (about 6 times more frequently). Ossicular chain reconstruction on intact stapes (assessed by SAMEO-ATO-O_2_) was associated with a lower complication prevalence than other ossicular chain situations (about 92% less frequently).

**Table 3:** Regression model estimates

Predictor		Univariate analysis		Multivariate analysis	
		OR	*p* value	OR	*p* value
Age	Each year increase	1.06 (1.02–1.10)	0.003*	1.07 (1.02, 1.12)	0.007*
Gender	Female vs male	0.31 (0.09–0.89)	0.038*	0.18 (0.03, 0.79)	0.030*
Side	Right vs left	1.71 (0.61–4.78)	0.303		
STAMCO-S	More than 0 vs 0	2.25 (0.78–7.02)	0.142		
STAMCO-T	Yes vs no	4.07 (1.06–26.75)	0.073**	6.94 (1.02, 73)	0.057**
STAMCO-A	Yes vs no	1.95 (0.49–13.01)	0.402		
STAMCO-M	Yes vs no	3.86 (1.34–12.89)	0.017*		
STAMCO-O	2 or 3 vs 1 or *n*	1.60 (0.51–5.28)	0.423		
ChOLE-Ch	3 or 4a vs lower	2.96 (1.04–8.48)	0.041*		
ChOLE-O	Each step increase	2.25 (0.51–9.09)	0.252		
ChOLE-E	Yes vs no	4.00 (0.57–34.42)	0.163		
SAMEO-ATO S	2p or 2r vs 1	1.26 (0.44–3.49)	0.661		
SAMEO-ATO A_1_	4 vs 1, 2, or 3	1.84 (0.31–35.13)	0.574		
SAMEO-ATO M	2c/3a vs 0/1a/1b/2a/2b	1.03 (0.37–2.95)	0.073**		
SAMEO-ATO O_1_	2 vs 1	13.0 (1.21–323)	0.052**	6.03 (1.19–35)	0.034*
SAMEO-ATO A_2_	2,3 vs 1, x	1.26 (0.46–3.71)	0.657		
SAMEO-ATO T	3 vs 1 or 2	3.17 (1.02–10.06)	0.045*	5.4 (1.11–32)	0.044*
SAMEO-ATO O_2_	S- vs F-	0.10 (0.00–0.69)	0.053**	0.08 (0–0.59)	0.036*

*Significative, **close to significance level

At univariate analysis, mastoid involvement (assessed with STAMCO-M and ChOLE-Ch) appeared to be related to complication finding.

NIR was 0.85 and the final model accuracy was 0.92 (95% CI 0.85, 0.97), significantly higher than NIR (0.028). The NPV was 89%, the PPV was 92%. ChOLE-L and STAMCO-C were excluded from the multivariable analysis, since they represent themselves pre- and peri-operative complication. ChOLE-O and ChOLE-E were also excluded from the multivariable analysis due to their high missingness frequency.

## Discussion

Middle ear surgery recognises many surgical variants, like surgical approach or ossicular chain reconstruction, and this leads to wide heterogeneity comparing data series. Recently, a commendable effort was made to create a common language about middle ear and temporal bone surgery for cholesteatoma [2, 3, 5, 6]. In our data, surgical procedures performed at our centre for middle ear cholesteatoma were retrospectively evaluated, and surgical records were recorded according to STAMCO, ChOLE, and SAMEO-ATO criteria. The focus of our analysis was to identify any relationship between pre- or intra-operative complications and surgical variants or anatomic areas involved by pathology.

Statistical analysis pointed out a protective role of female gender in the occurrence of intra-operative complications [*p* = 0.03, OR 95% CI 0.18 (0.03–0.79)], in accordance with the literature data [8]. Patient age appeared to be correlated with an increased risk of intra-operative complication [*p* = 0.004, OR 95% CI 1.07 (1.02–1.12)]. Age of 49 years was assessed as a cut-off value for the increased risk of surgical complications [95% CI (95% CI 0.628–0.864)] in accordance with the previous literature reports [9]. Univariate analysis pointed out an association between mastoid cavity involvement and intra-operative complication, assessed with both STAMCO-M [*p* = 0.017, 95% CI 3.86 (1.34–12.89)] and ChOLE-Ch [*p* = 0.041, 95% CI 2.96 (1.04–8.48)].

At multivariate analysis, tympanic cavity involvement accessed by STAMCO classification (STAMCO-T) was associated with an increase of surgical complication rate of 7 times [*p* = 0.06, 95% CI 6.94 (1.02–73)]. Intraoperative necessity of a total or subtotal tympanic membrane grafting was associated with an increased complication prevalence of 5.4 times compared with patients which underwent a partial tympanic membrane reconstruction (*p* = 0.04, OR 95% CI 5.40 (1.11–32). Mastoid obliteration (SAMEO-ATO-O_1_) appeared to be related to an increased prevalence of intra-operative complication finding (*p* = 0.03). In our study, ossicular chain reconstruction performed on stapes head was less frequently associated with intra-operative complication findings [*p* = 0.04, OR 95% CI 0.08 (0.00–0.59)].

Association between necessity of total tympanic membrane grafting and complication finding can be explained by a more widespread pathology at the time of surgery. In our data series, almost every complication was related to mastoid cavity involvement, and this finding could explain the correlation between mastoid involvement (assessed with STAMCO-M and ChOLE-Ch) and surgical complications. The lack of a confirmation at multivariate analysis of correlation between surgical complications and mastoid involvement (STAMCO-M and ChOLE-Ch) could be related to the fact that mastoid cavity is considered as a single unit, while there are areas at higher risk of complications than others. For example, peri-labyrinthine cells area or cranial portion of the mastoid (next to tegmen antri) involvement could lead to a higher risk of LSC fistula and middle cranial fossa fistula respectively, compared to the mastoid apex area.

Takahashi et al. pointed out that bone density plays an important role in spreading pattern of acute mastoiditis subsites [10]. In our opinion, cholesteatoma spreading could be considered, similarly to acute mastoid infections, related to different bone density in mastoid, even though this data cannot be further analysed in this data series.

Mastoid obliteration is usually performed when a canal wall down mastoidectomy is required, to reduce mastoid cavity volume. This trick favours surgical mastoid cavity shrinking, thus reducing wax deposit and risk of otorrhea [11]. In our data series, mastoid obliteration is indicator of a more extended surgery due to extension of cholesteatoma. Moreover, in a recent review, Young et al. reported that mastoid obliteration is a viable option in cholesteatoma surgery and is not associated with an increased risk of residual or recurrent cholesteatoma [12]. Ossicular chain erosion is a common finding in middle ear surgery for chronic otitis media with cholesteatoma. Up to 82% of patients could present with an ossicular erosion and the most involved ossicle is the incus [13]. Association between ossicular chain reconstruction performed on intact stapes and lower intra-operative complication rate could be explained by a more limited cholesteatoma, that anatomically is located far from labyrinth, the most common site of complications [14, 15].

Labyrinthine fistula is one of the most frequent complication in chronic otitis with cholesteatoma. The most frequent subsite involved is LSC, ranging from 4.5% [14] to 16.7% [15]. In our case series, LSC fistulas occurred in 7.6% of cases, in line with the literature data.

The main limitations of our study are represented by the small number of patients and the retrospective nature of the study which does not allow us to provide definitive results. Further prospective studies on larger cohort of patient are required to confirm our results.

The prediction model was not validated on external data source, and thus is likely to overfit the study observations. Validation on a different cohort of patients is required to confirm the current exploratory findings. ChOLE-O and ChOLE-E were excluded from the multivariable analysis due to their high missingness frequency.

## Conclusion

Classification of chronic otitis with cholesteatoma has been a matter of debate until now. Recent classifications of middle ear and petrous bone cholesteatomas could lead to a uniformity in surgical reports and wider data series comparison. The choice of variables to be recorded for cholesteatoma staging should be carefully balanced, considering that “complication” variable could be a repetitive item. Our aim is to critically analyse the relationships between complications and the other STAMCO and ChOLE classifications variables. STAMCO and ChOLE classifications could represent a novel “TNM” for cholesteatoma surgery and, in our opinion, complication variable (STAMCO-C or ChOLE-L) is a topic to go further on. SAMEO-ATO classification is a useful classification that includes the main middle ear surgical variables and allows a retrospective data collection as well as a prospective data set.

In our series, statistical analysis pointed out a relationship between surgical complications and age, gender, cholesteatoma site, mastoidectomy type, and ossicular chain status at surgery. Due to the small number of patients involved and the retrospective nature of the study, larger cohort of patients and prospective studies are required to confirm our results.
